# Co-infection dynamics of *B. afzelii* and TBEV in C3H mice: insights and implications for future research

**DOI:** 10.1128/iai.00249-24

**Published:** 2024-07-11

**Authors:** Stefania Porcelli, Aurélie Heckmann, Pierre Lucien Deshuillers, Alejandra Wu-Chuang, Cleménce Galon, Lourdes Mateos-Hernandez, Sabine Rakotobe, Laetitia Canini, Ryan O. M. Rego, Ladislav Simo, Anne-Claire Lagrée, Alejandro Cabezas-Cruz, Sara Moutailler

**Affiliations:** 1 ANSES, INRAE, Ecole Nationale Vétérinaire d’Alfort, UMR BIPAR, Laboratoire de Santé Animale, Maisons-Alfort, France; 2 EPIMIM, Laboratoire de Santé Animale, Anses, Ecole Nationale Vétérinaire d’Alfort, Maisons-Alfort, France; 3 Institute of Parasitology, Biology Centre, Czech Academy of Sciences, Ceske Budejovice, Czechia; 4 Faculty of Science, University of South Bohemia, Ceske Budejovice, Czechia; University of California Davis, Davis, California, USA

**Keywords:** co-infection, tick-borne pathogens, C3H mice, pathogen fitness, pathogen invasiveness

## Abstract

Ticks are important vectors of disease, particularly in the context of One Health, where tick-borne diseases (TBDs) are increasingly prevalent worldwide. TBDs often involve co-infections, where multiple pathogens co-exist in a single host. Patients with chronic Lyme disease often have co-infections with other bacteria or parasites. This study aimed to create a co-infection model with *Borrelia afzelii* and tick-borne encephalitis virus (TBEV) in C3H mice and to evaluate symptoms, mortality, and pathogen level compared to single infections. Successful co-infection of C3H mice with *B. afzelii* and TBEV was achieved. Outcomes varied, depending on the timing of infection. When TBEV infection followed *B. afzelii* infection by 9 days, TBEV symptoms worsened and virus levels increased. Conversely, mice infected 21 days apart with TBEV showed milder symptoms and lower mortality. Simultaneous infection resulted in mild symptoms and no deaths. However, our model did not effectively infect ticks with TBEV, possibly due to suboptimal dosing, highlighting the challenges of replicating natural conditions. Understanding the consequences of co-infection is crucial, given the increasing prevalence of TBD. Co-infected individuals may experience exacerbated symptoms, highlighting the need for a comprehensive understanding through refined animal models. This study advances knowledge of TBD and highlights the importance of exploring co-infection dynamics in host-pathogen interactions.

## INTRODUCTION

Ticks, second only to mosquitoes, are well-known carriers of disease-causing agents (1). This concern has grown under the One Health approach, with tick-borne diseases (TBDs) increasingly prevalent worldwide (2). For instance, the Centers for Disease Control and Prevention reported 50,865 TBD cases in the USA, in 2019. Among these, the most prevalent illnesses were Lyme disease (LD) (34,945 cases) and anaplasmosis/ehrlichiosis (7,976 cases). In the USA, the most commonly documented human co-infection is between Lyme disease and babesiosis (3). On the other hand, in Europe, tick-borne encephalitis frequently co-exists with Lyme disease (3, 4). However, comparatively, researchers in both Europe and the USA have extensively studied ixodid ticks for co-infections with important pathogens such as *Borrelia burgdorferi sensu lato* (s.l.), *Anaplasma phagocytophilum*, *Babesia microti*, and *Rickettsia* spp. In Europe, these co-infections mainly consist of combinations such as *B. burgdorferi* s.l., *A. phagocytophilum*, and *Ba. microti*, or *B. burgdorferi* s.l. with *A. phagocytophilum*, or *A. phagocytophilum* with *Rickettsia* spp. In the USA, co-infections are mainly with *B. burgdorferi sensu stricto* (s.s.) together with either *A. phagocytophilum* or *Ba. microti* (3).

Tick-borne encephalitis virus (TBEV), a member of the flavivirus family, is transmitted by ticks or through consuming infected raw goat milk or unpasteurized dairy products, leading to localized outbreaks (5). While TBEV often causes fever, it can progress to neurological symptoms (6). To date, five TBEV subtypes have been described based on phylogenetic clustering and geographical distribution, although different subtypes can be found in each endemic region in different proportions. TBEV subtypes include Western (European; TBEV-Eu), Siberian (Eastern; TBEV-Sib), Far-Eastern (TBEV-Fe), Baikalian (TBEV-Bkl), and Himalayan (TBEV-Him) (7 – 9).

On the other hand, LD is caused by bacteria of the *Borrelia* genus. Within this genus, at least three genospecies of the *B. burgdorferi* s.l. complex are known to affect humans in Europe: *Borrelia afzelii*, *B. garinii*, and *B. burgdorferi* s.s. LD is a multi-phased syndrome affecting skin, muscles, bones, and nerves (10). Diagnosing these diseases can be challenging due to their non-specific symptoms and the potential for co-infections.

Co-infection, whereby multiple pathogen species or genotypes co-exist within the same host (11), with TBDs is not rare. Serological studies show that patients with chronic Lyme disease often have one (23.5%) or more (30%) co-infections with other bacteria or parasites (12). However, detecting multiple infections is difficult due to overlapping symptoms and varying incubation periods, making its diagnosis and study challenging.

Research using murine models (mostly C3H/HeN mouse strain) has extensively documented the pathogenesis of Lyme disease and TBEV single infection (13, 14). Studies also show that co-infection with *B. burgdorferi* s.s. and *A. phagocytophilum* alters the immune response and affects Lyme arthritis severity (15, 16). However, in general, there is a lack of models studying tick-borne co-infections with a virus and a bacterium. Such models could help in understanding the interactions between the two infectious agents and their impact on the associated disease, as well as guide therapy (17).

Infections with different diseases can arise from multiple ticks or a single tick carrying multiple pathogens (18).

Hence, our study aims to (i) establish a *B. afzelii*/TBEV co-infection model in C3H mice; (ii) examine symptoms, mortality, targeted organs, and pathogen level compared to single infections; (iii) determine if the pathogens have synergistic or antagonistic effects when infecting at different times; and (iv) explore pathogen transmission from co-infected mice to uninfected ticks.

In an attempt to reproduce different natural infection scenarios, the mice were divided into different infection groups, taking into account the lethal effect of TBEV on mice within 12 days (19) and the 20 days required for *B. afzelii* antibodies to be produced in the mice (20). The super-infection 1 (S1) group consisted of mice first infected with *B. afzelii* and after 20 days with TBEV; the co-infection (co-inf.) group consisted of the simultaneous infection of the pathogens; and finally, the super-infection 2 (S2) group consisted of mice infected first with *B. afzelii* and 9 days later with TBEV. Because of the high lethality of TBEV infections, this last group was designed as an alternative to the co-infection group in which *B. afzelii* may not have the time to spread before mice death or euthanasia related to TBEV. Therefore, since *B. afzelii* did not induce symptoms in C3H mice (20), the assessment of clinical signs and pathogen levels in different organs started after the TBEV infection.

## MATERIALS AND METHODS

### Mice and housing conditions

Six-week-old female C3H/HeN (C3H) mice were obtained from Charles River Laboratories (France). The mice were maintained in groups of five, or individually when infested with ticks, in plastic cages with wood-chip bedding, fed *ad libitum*, and kept in standardized conditions (21°C, 12-hour light/12-hour dark photoperiod) at the accredited animal facilities of the French Agency for Food, Environmental and Occupational Health & Safety in Maisons-Alfort, France. Mice were monitored twice daily, documenting and reporting any deviations from normal behavior or signs of health decline. Animal housing and handling took place under biosafety level 3** conditions.

### Bacterial culture

Low passage of *B. afzelii* CB43 was started from glycerol stocks and grown in Barbour-Stoenner-Kelly-H (BSK-H) (Sigma-Aldrich, St. Louis, MO, USA) medium with 6% rabbit serum at 33°C for 7 days after being started from glycerol stocks.

### Viral culture

Mice were infected with TBEV strain Hypr. This strain was isolated from human blood in the Czech Republic in 1953 (21). TBEV Hypr was passaged four times in suckling mice brains and twice in Vero E6 cells and was used in the experiments. The viral production had a titer of 2.3 × 10^6^ PFU/mL.

### Ticks

*Ixodes ricinus* larval ticks were obtained from a colony of the Institute of Zoology, Slovak Academic of Sciences, Bratislava Slovakia. The ticks were maintained in an incubator with a temperature of 21°C and 95% humidity exposed to a 12-hour light/12-hour dark photoperiod.

### Experimental infection of mice

**Fig 1:**
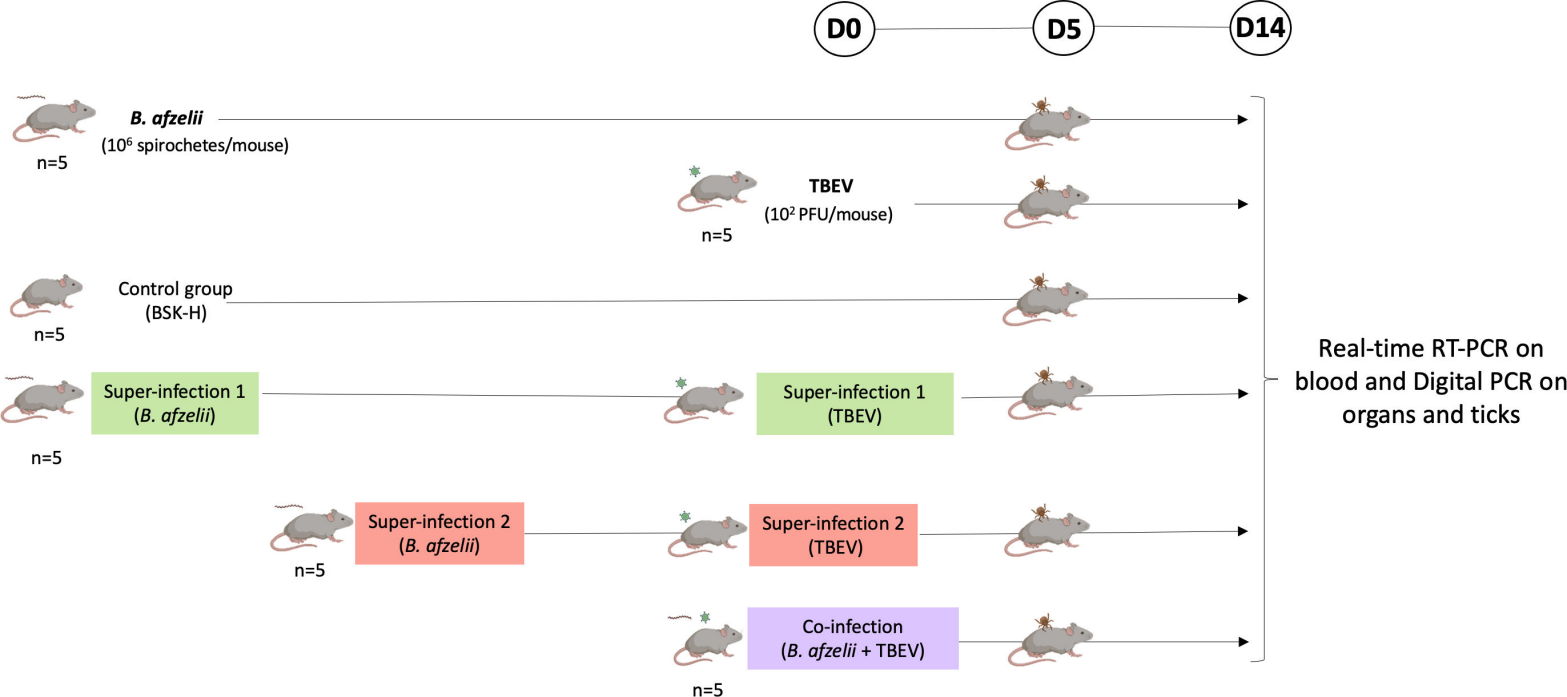
Representation of the experimental design. Infection of C3H mice at different time points. *Borrelia afzelii*, mice infected with 1 × 10^6^ spirochetes/mouse (*n* = 5); TBEV, mice infected with 1 × 10^2^ PFU/mouse (*n* = 5); control group, mice inoculated with 100 µL of BSK-H medium (*n* = 5); super-infection 1, mice infected with *B. afzelii* followed by TBEV after day 21 (*n* = 5) (same doses than single infection); super-infection 2, mice infected with *B. afzelii* followed by TBEV after 9 days (*n* = 5) (same doses than single infection); co-infection, mice infected simultaneously with *B. afzelii* and TBEV (*n* = 5) (same doses than single infection). Acquisition of both pathogens by ticks was assessed by placing naïve larvae at day 5 p.t.i. on the back of two mice per group. The infection was monitored by collecting blood samples on days 0, 5, 8, and 12 p.t.i., and mice still alive were sacrificed on day 14 p.t.i. to collect organs. Blood samples were tested for TBEV viral RNA using real-time reverse transcription PCR (RT-PCR). Organs and ticks were tested for both pathogens using digital PCR after undergoing reverse transcription and pre-amplification procedures to quantify the copy number of each pathogen.

After a 1-week acclimatization period, 30 mice were divided into six study groups of five mice each: *B. afzelii*, TBEV, S1, in which mice were infected with *B. afzelii* followed by TBEV after 21 days; S2, in which mice were infected with *B. afzelii* followed by TBEV after 9 days; a co-infection group, in which mice were infected with both pathogens simultaneously; and a negative control group. Mice were infected with TBEV via subcutaneous injection of the Hypr strain viral suspension (100 µL), with 1 × 10^2^ PFU/mouse. In the *B. afzelii* group, mice were infected with 1 × 10^6^ spirochetes of *B. afzelii* (CB43 strain) through a combination of subcutaneous (100 µL) and intraperitoneal (150 µL) injections in BSK-H medium (Sigma-Aldrich) (20). Control mice received 100 µL of BSK-H medium subcutaneously. Over 14-day post-TBEV infection (p.t.i.) observation period, TBEV-specific symptoms such as ruffled fur, hunched posture, paralysis, and mortality were documented for each mouse. When severe clinical signs of TBEV were observed, mice were euthanized before the endpoint of the study. At the endpoint, various organs (brain, spleen, heart, joint, bladder, and ear) were gathered and stored at −80°C until further use (Fig. 1).

### Ticks’ infestation

On day 5 p.t.i., mice that were previously infected were exposed to pathogen-free ticks. This was carried out to document the acquisition of *B. afzelii* and/or TBEV from the mice. For that, 100 naïve larvae of pathogen-free unfed *I. ricinus* were placed in a capsule system on the back of 12 mice (2 mice from each of the six groups) until repletion as described before (22). In this study, we used a system with a capsule attached to a laboratory mouse to create a feeding device tailored to the nymphal and larval stages of hard tick (23). Fifteen to 20 fully engorged larvae from each group were conserved at −80°C until further use, and the remaining larvae were stored in an incubator until molting. After the molt, 5–10 nymphs from each group were sacrificed and stored at −80°C until further use.

### Blood sample collection and preparation

Blood samples (50 µL) were collected from each mouse on days 0, 5, 8, and 12 p.t.i through retro-orbital sampling from all experimental groups in sterile tubes with 30 µL EDTA. Subsequently, 50 µL of blood were combined with 350 µL of lysis buffer (RA1) and 3.5 µL of β-mercaptoethanol in tubes, using the NucleoSpin RNA extract II kit (Macherey Nagel, Germany). These tubes were then frozen at −80°C until RNA extraction.

For sera collection, blood samples obtained on days 8 and 11 p.t.i were allowed to clot for 2 hours at room temperature. After clotting, they were centrifuged twice at 5,000 × *g* for 5 min at room temperature. The resulting sera were separated and placed into new sterile tubes, which were stored at −20°C for subsequent Western blot analysis.

### Bacterial protein extraction

Lysates of *B. afzelii* culture were prepared to perform Western blots. Seven milliliters of culture of *B. afzelii* with a density of at least 1 × 10^7^/mL were centrifuged at 8,000 rpm for 10 min at 20°C. The supernatant was then removed, and the bacterial pellet was washed twice with 1-mL cold HN buffer, and centrifuged at 8,000 rpm for 10 min at 20°C. The resulting pellet was resuspended in 200 µL of bacterial protein extraction buffer (Thermo Scientific, Waltham, MA, USA) and incubated at room temperature for 10 min. Protein concentration was determined using the Bradford Protein Assay (Thermo Scientific, San Jose, CA, USA) with bovine serum albumin (BSA) as standard, and the lysate was stored at −20°C until use.

### Western blot

The evaluation of the immune response against *B. afzelii* infection in mice was conducted using Western blot analysis. Twenty micrograms from lysates of *B. afzelii* was combined with an equal volume of 2× Laemmli buffer (Thermo Scientific) and heat-denatured at 100°C for 10 min. Prepared lysates were loaded onto a 4%–15% Mini-PROTEAN TGX Stain-Free Protein gel (Bio-Rad, Hercules, CA, USA), and SDS-PAGE electrophoresis was conducted at 120 V for 1 hour.

Subsequently, proteins were transferred to a nitrocellulose membrane (Bio-Rad) using a semi-dry transfer method. Blotting took place for 10 min at 25 V in a transfer cell (Trans-Blot SD, Bio-Rad). The membrane was blocked then with 1% BSA/phosphate-buffered saline (PBS) (Sigma-Aldrich) for 2 hours at room temperature, followed by overnight incubation with mouse sera at a 1:100 dilution in PBS at 4°C.

On the following day, the membranes were washed three times for 10 min each in PBS Tween 0.005% with gentle rocking. Subsequently, the membranes were exposed to horseradish peroxidase-conjugated antibodies (goat anti-mouse IgG) (Sigma-Aldrich) at a 1:2,000 dilution in PBS for 1 hour at room temperature with gentle shaking. After three washes, antibody detection was performed through chemiluminescence using Pierce Enhanced chemiluminescence (ECL) Western blotting substrate (Bio-Rad). The membranes were treated with ECL reagent for 3 min, and images of the membranes were captured using the ChemiDoc Touch Imaging System (Bio-Rad).

### DNA extraction

NucleoSpin tissue DNA extraction Kit (Macherey Nagel) was used for mouse tissue genomic DNA extraction. The tissues (1/2 brain, 1/2 spleen, ½ heart, one joint, one ear, and 1/2 bladder) were crushed utilizing the FAST PREP-24 5G equipment from MP Biomedicals, USA. To achieve this, each individual organ was homogenized in 500 µL of DMEM (Fisher Scientific, France) supplemented with 10% Fetal Bovine Serum (FBS) using a homogenizer (MP Biomedicals) equipped with six stainless-steel beads. The homogenization process consisted of 3 × 20 seconds-long cycles, followed by a 5 min centrifugation step at 10,000 × *g*. Subsequently, DNA was extracted from 250 µL of the homogenate employing the NucleoSpin DNA extraction kit. The resulting DNA was eluted in 40 µL of DNase-free water and stored at −20°C.

### RNA extraction

RNA was isolated from blood samples (30 µL) using the NucleoSpin RNA extract II kit (Macherey Nagel) following the manufacturer’s instructions. The eluted RNA samples were stored at −80°C. For each organ, RNA was extracted from 250 µL of the homogenate obtained (previously described in DNA Extraction) using the NucleoSpin RNA extract II kit, and RNA was eluted in 40 µL of RNase-free water and stored at −80°C.

For RNA extraction from ticks, individual specimens were homogenized in 350 µL of lysis buffer, along with 3.5 µL of β-mercaptoethanol. The homogenization was achieved using the same homogenizer (MD Biomedical) with six stainless-steel beads, undergoing two cycles of 20 seconds each at 4-min intervals. Following homogenization, the samples were subjected to centrifugation for 5 min at 10,000 × *g*. RNA was eluted with 30 µL of RNase-free water and stored at −80°C before further use.

### RNA reverse transcription

To generate cDNA from mouse organs to detect TBEV and from ticks to target *B. afzelii* RNA and TBEV, the Reverse Transcription (RT) Master Mix (Standard Biotools, San Francisco, USA) was employed, followed by pre-amplification using the Fluidigm Preamp Master Mix (Standard Biotools) following the manufacturer’s guidelines.

For the Reverse Transcription Master Mix, a 5× master mix was employed, combining 1 µL of Reverse Transcription Master Mix, 3 µL of RNase-free water, and 1 µL of RNA. The reverse transcription process included thermocycler cycles at 25°C (5 min), 42°C (30 min), and 85°C (5 min).

### DNA and cDNA pre-amplification

The DNA and cDNA samples (after undergoing reverse transcription) were either stored at −20°C or utilized immediately for pre-amplification reactions using the Preamp Master Mix kit (Standard Biotools). During the pre-amplification step, primers targeting *B. afzelii* and TBEV were pooled equally at a final concentration of 0.2 µM each. The following primers used to target *B. afzelii* and TBEV are shown in Table 1.

**TABLE 1:** Oligonucleotide primers used to detect tick-borne pathogens

Species	Primer	Sequence (5′→3′)	Target gene [size (bp)]	Reference
*Borrelia afzelii*	Bo_af_fla_F Bo_af_fla_R Bo_af_fla_P	GGAGCAAATCAAGATGAAGCAAT TGAGCACCCTCTTGAACAGG TGCAGCCTGAGCAGCTTGAGCTCC	*fla* (116)	(24)
Tick-borne encephalitis virus European subtype	TBE Euro F TBE Euro R TBE Euro *P*	TCCTTGAGCTTGACAAGACAG TGTTTCCATGGCAGAGCCAG TGGAACACCTTCCAACGGCTTGGCA	E (91)	(25)

The pre-amplification reactions took place in a total volume of 5 µL, composed of 1 µL of Preamp Master Mix, 1.25 µL of the pooled primer mixture, 1.5 µL of distilled water, and 1.25 µL of cDNA.

The PCR conditions included an initial denaturation step at 95°C (2 min), followed by 14 amplification cycles at 95°C (15 s) and 60°C (4 min). Subsequently, the pre-amplified products were subjected to various dilutions (1:2, 1:5, 1:100, 1:500, and 1:1,000) with distilled and sterile water, and they were stored at −20°C for future utilization.

### Real-time RT-PCR for TBEV detection in the blood

Real-time RT-PCR was carried out on blood samples utilizing the LightCycler 480 RNA Master Hydrolysis Probes kit (Roche Diagnostics, Germany) as per the manufacturer’s guidelines. The RT-PCR reaction volume was 20 µL, consisting of 7.4 µL of LightCycler 480 RNA Master Hydrolysis Probes, 8.05 µL of water, 1.3 µL of activator, 2 µL of primers and probes for detecting *B. afzelii* and TBEV, and 2 µL of RNA template.

The following primers and probe used to detect TBEV are described in Table 1.

The real-time RT-PCR parameters consisted of a reverse transcription cycle at 63°C for 3 min, followed by a denaturation cycle at 95°C for 30 s, 45 successive cycles at 95°C for 10 s, 60°C for 30 s, and 72°C for 1 s, and a final step of cooling at 40°C for 30 s.

### Quantification of *B. afzelii* and TBEV by digital PCR on the BioMark System

Digital PCR (dPCR) amplifications were conducted on mice organs and ticks using the with quantitative dPCR (qdPCR) 37 k integrated fluidic circuit (IFC) digital array microfluidic chips (Standard Biotools). The BioMark IFC controller utilized nanoscale valves and channels to partition each of the 48 samples, pre-mixed with PCR reagents, into a panel of 770 PCR reaction chambers, resulting in a total of 36,960 individual qdPCR reactions on the digital array. The number of target molecules in each sample was accurately estimated by counting the positive reactions, based on the Poisson distribution.

Each reaction mixture consisted of a 6-µL total volume consisting of 3 µL of 2× Perfecta qPCR tough mix along with ROX reference dye from Standard Biotools, 0.6 µL of 20× GE Sample Loading Reagent also from Standard Biotools, 0.3 µL of 20× primer stock containing 100-µM concentration of each forward and reverse primers, 20 µM of TBEV and *B. afzelii* probes, and 1.8 µL of pre-amplified cDNA sample. The experiments included a positive control containing cDNA extracted from a viral suspension and a bacterial culture and a negative control containing water.

For loading into the chip with the IFC controller MX, 4 µL out of the 6 µL reaction mix was used, and 0.65 µL was effectively partitioned into the 770 chambers of one panel, including 0.38 µL of cDNA extract. The qdPCR program consisted of 2 min at 50°C and 10 min at 95°C, then 40 cycles where the samples were subjected to 95°C for 15 s and 60°C for 60 s. Fluorescence was recorded by the apparatus at the end of each elongation step (1 min at 60°C) for every amplification cycle. The Digital PCR Analysis software, a component of the BioMark System (Standard Biotools), was employed to count the number of positive chambers out of the total number of chambers per panel. The Poisson distribution was used to estimate the average number of template copies per chamber in a panel. Each sample was characterized by its corresponding absolute quantity, and no positive chambers were observed in negative samples.

### Statistical analysis

The differences in survival time of infected mice were analyzed by survival analysis (long-rank Mantel-Cox test). The analyses were performed using GraphPad Prism version 10 (GraphPad Software); *P* values of <0.05 were considered significant.

## RESULTS

### Establishing a co-infection model with *B. afzelii* and TBEV in C3H mice

To develop a model of co-infection with *B. afzelii* and TBEV, C3H mice underwent various infection scenarios (Fig. 1). Throughout the study, infections were identified in two ways: directly, by using real-time RT-PCR to detect TBEV in blood samples, and indirectly, by finding antibodies against *B. afzelii* in the mice’s sera. In the TBEV group, only two out of five mice showed infection at 8 days p.t.i. In the S1 group, all mice had detectable TBEV RNA at 8 days p.t.i. In the S2 group, all mice were positive for TBEV RNA detection between 5 and 8 days p.t.i. In the co-inf. group, TBEV RNA was detected in four out of five mice at different time points (Table 2).

**TABLE 2:** TBEV RNA detection in the blood of C3H-infected mice by real-time RT-PCR

Days post-TBEV infection	D0^*b*^	D5	D8	D12
Negative control	0/5^*a*^	0/5	0/5	0/5
*B. afzelii*	0/5	0/5	0/5	0/5
TBEV	0/5	1/5	2/5	0/3
Super-infection 1	0/5	4/5	5/5	4/4
Super-infection 2	0/5	5/5	5/5	3/3
Co-infection	0/5	4/5	4/5	4/5

^*a*^ The number of positive mice/number of tested mice.

^*b*^ D, days.

*B. afzelii* infection was confirmed by Western blot analysis using mice sera against *B. afzelii* protein extracts. On day 8 p.t.i, bands corresponding to specific antigen-antibody interactions were observed in all infected groups, indicating the development of antibodies against *B. afzelii* antigens. Notably, the intensity of the bands varied across infection groups, suggesting potential differences in the magnitude or kinetics of the immune response (Fig. S1).

### Symptoms of C3H-infected mice with *B. afzelii* and TBEV and their survival curve

Initially, distinct clinical manifestations typical of TBEV infection, such as ruffled hair, hunched posture and paralysis, occurred in the TBEV, S1, S2, and co-inf. groups. Among the TBEV-infected mice in the TBEV group, three out of five did not show any TBEV symptoms, while two out of five showed all symptoms.

In the S1 group, all five mice developed all TBEV symptoms. In contrast, all five mice in the co-inf. group showed mild symptoms, including ruffled hair, two mice out of five with a hunched posture, and none with paralysis (Table 3).

**TABLE 3:** Symptoms of C3H-infected mice with *Borrelia afzelii* and TBEV

	No symptoms	Ruffled hair	Hunched posture	Paralysis
Negative control	5/5^a^	0/5	0/5	0/5
*B. afzelii*	5/5	0/5	0/5	0/5
TBEV	3/5	2/5	2/5	2/5
Super-infection 1	0/5	5/5	5/5	5/5
Super-infection 2	1/5	4/5	4/5	4/5
Co-infection	0/5	5/5	3/5	0/5

^a^ The ratio of mice exhibiting symptoms/total number of tested mice.

**Fig 2:**
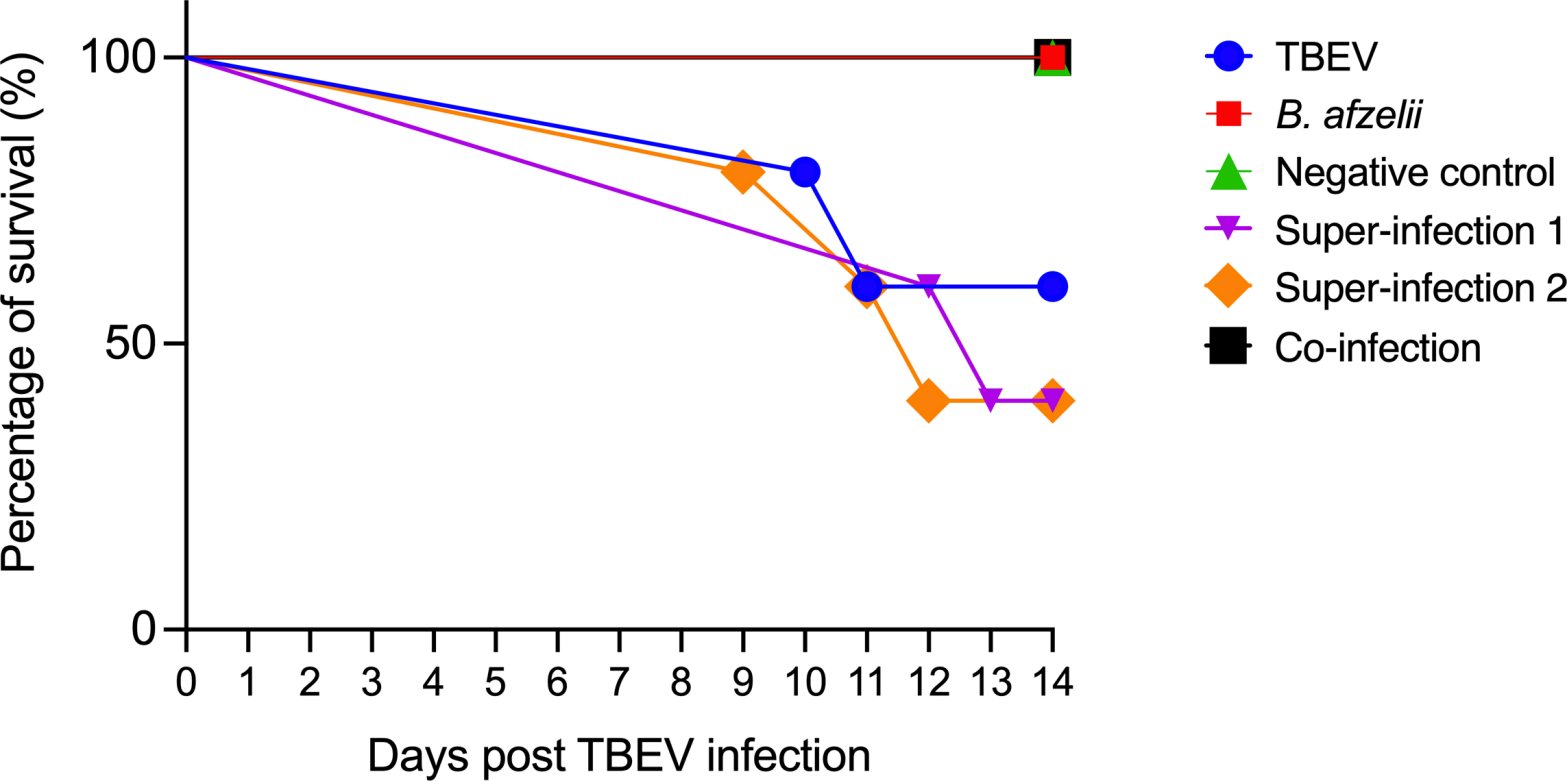
Survival curve of C3H-infected mice. The mice were exposed to different infection scenarios (*n* = 5 in each group). TBEV, 10^2^ PFU; *Borrelia afzelii*, 10^6^ spirochetes; super-infection 1, mice infected with *B. afzelii* followed by TBEV after 21 days; super-infection 2, mice infected with *B. afzelii* followed by TBEV after 9 days; co-infection, mice infected with *B. afzelii* and TBEV simultaneously; and one negative control group inoculated with BSK-H medium.

However, a survival curve was recorded. While all mice in the negative control, *B. afzelii*, and co-inf. groups survived until the end of the experiment; 60% of mice in the S1 and S2 groups and 40% of mice in the TBEV group died following TBEV infection. In particular, in the TBEV group, two out of five died at 10 and 11 days p.t.i., respectively. Conversely, in the S1 group, three out of five also died between 12 and 13 days p.t.i. In the S2 group, mortality occurred at 9 days p.t.i with two additional deaths recorded 11 and 12 days p.t.i. Survival curve analysis showed statistically significant differences between the S1, S2, co-inf., *B. afzelii*, TBEV, and negative control groups, as confirmed by the log-rank Mantel-Cox test (*P* < 0.05) (Fig. 2).

### Quantification of *B. afzelii* and TBEV in organs of C3H-infected mice in different conditions

During the 14-day observation period following TBEV infection, when severe clinical signs of TBEV were evident, the mice were euthanized before the endpoint of the study, allowing us to assess the levels of TBEV and *B. afzelii* in various organs of the C3H mice.

Table 4 displays the ratio of mice testing positive out of the total number analyzed in the respective organs across different conditions. In the TBEV infection group, it was consistently observed that three out of five mice showed negative TBEV RNA detection across all tested organs, aligning with previous observations of negative results in their blood samples and no symptoms observed.

**TABLE 4:** Detection of C3H-infected mice with *Borrelia afzelii* and TBEV in different organs

	Brain		Heart		Joint		Ear		Bladder		Spleen	
	B^*b*^	T	B	T	B	T	B	T	B	T	B	T
Single infection	1/5^*a*^	2/5	5/5	2/5	5/5	1/5	5/5	2/5	5/5	2/5	0/5	1/5
Super-infection 1	1/5	4/5	4/5	4/5	4/5	2/5	4/5	4/5	4/5	5/5	2/5	1/5
Super-infection 2	1/5	4/5	3/5	4/5	4/5	4/5	4/5	4/5	3/5	4/5	1/5	2/5
Co-infection	0/5	3/5	2/5	4/5	4/5	2/5	3/5	3/5	2/5	3/5	1/5	1/5

^*a*^ The number of positive mice/number of tested mice.

^*b*^ B, *B. afzelii*; T, TBEV RNA.

An unexpected finding arose with the detection of TBEV RNA in the ears and the joints of infected mice across all experimental groups. We also observed the presence of *B. afzelii* DNA in the brains of a small number of mice (one out of five) across the *B. afzelii*, S1, and S2 groups.

Furthermore, the spleen exhibited the lowest incidence of positive results for both pathogens. None of the mice in the *B. afzelii* group tested positive for *B. afzelii* DNA in the spleen, and similarly, in the co-inf. group, none of the mice tested positive for *B. afzelii* DNA in the brain (taking into account the shorter duration of the infection).

**Fig 3:**
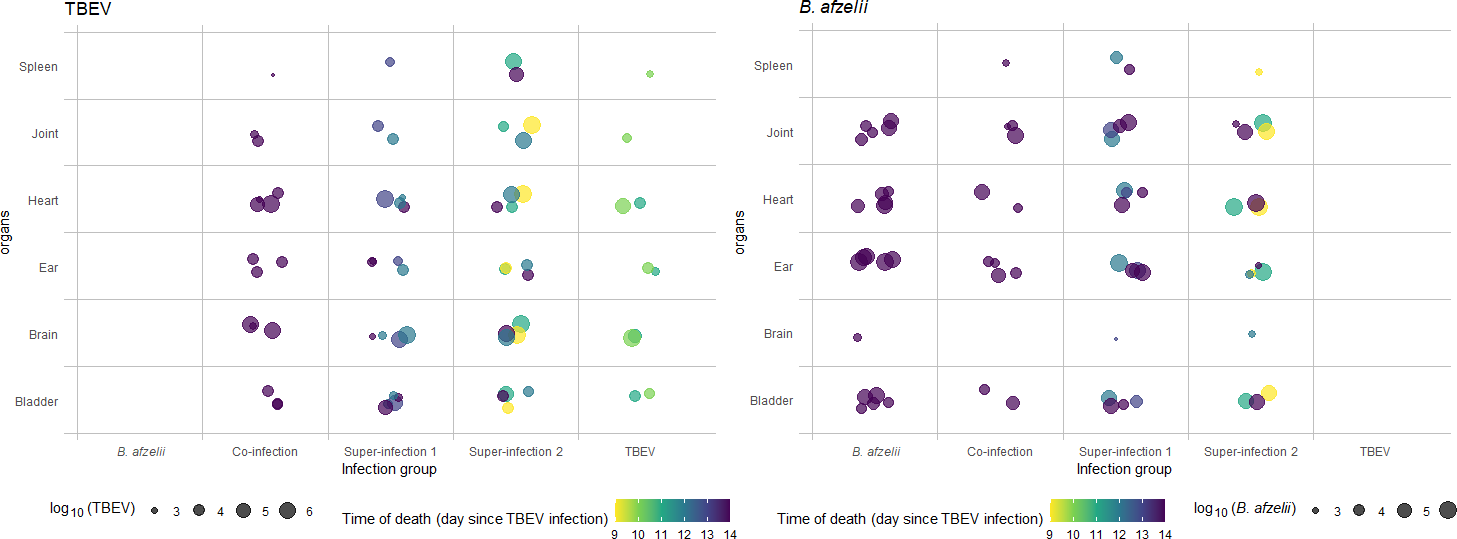
*B. afzelii* DNA and TBEV RNA quantification in the organs of C3H-infected mice by digital PCR. The graph illustrates the amount of pathogen (TBEV RNA or *B. afzelii DNA*) shed by individual mice relative to the time of death (using different colors) following TBEV infection, sorted by the specific combination of organ and infection group.

Subsequently, we analyzed the levels of each pathogen in every organ of each specific group. For example, in the TBEV group (where only two out of five mice tested positive), TBEV exhibited a higher level in the brain (averaging 10^6^ copies/µL) and heart (averaging 10^5^ copies/µL), compared to the urinary bladder, ear, joint, and spleen, in which the TBEV pathogen levels were lower (Fig. 3).

However, when focusing on the S1 and S2 groups, TBEV demonstrated a high level of pathogen in the brain (where four out of five mice tested positive), reaching even higher levels (10^6^ copies/µL) in the S2 group, where mice started to die at 9 days p.t.i. In addition, in the S1 group, the organs most affected were the brain, heart, and bladder (averaging 10^5^ copies/µL), but the mice started to die 12 days p.t.i. (Fig. 3).

Furthermore, in the S2 group, besides the brain and heart (averaging 10^6^ and 10^5^ copies/µL, respectively), TBEV also invaded the joint, which is not typically targeted by the virus, averaging 10^5^ copies/µL. Similar results were observed in the spleen (averaging 10^5^ copies/µL), which was the organ most affected by the virus compared to the other groups (Fig. 3).

Finally, in the co-inf. group, even if the TBEV levels were highest in the heart and brain (averaging 10^5^ copies/µL), followed by the ear, bladder, joint, and spleen (Fig. 3), the mice survived until the endpoint of the experiment.

Looking at the pathogen level of *B. afzelii* in the single infection group, we found that the primary organ affected was the ear (averaging 10^5^ copies/µL), followed by the heart, bladder, joint, and brain (Fig. 3). Notably, *B. afzelii* did not colonize the spleen in this group.

A similar trend was observed in the S1 group, although *B. afzelii* managed to colonize the spleen as well (averaging 10^3^ copies/µL).

Furthermore, in the S2 group, even though the infection with *B. afzelii* was shorter compared to the S1 and *B. afzelii* groups, the bacterium exhibited a distinct invasion pattern, with the highest degree of infiltration observed in the heart (averaging 10^5^ copies/µL), followed by the joint, ear, bladder, spleen, and brain (Fig. 3).

Finally, in the co-infected group, the presence of *B. afzelii* was reduced, mainly due to lower infection rates in the mice as a result of a shorter duration of infection. Consequently, the ear was the most affected organ, followed by the heart, joint, bladder, and spleen (Fig. 3).

### *Borrelia afzelii* and TBEV acquisition by naïve larvae in C3H mice

In this part of the experiment, conducted on day 5 p.t.i, we explored if naïve larvae (100 per mouse, with 2 mice in each group) acquire *B. afzelii* and TBEV from either singly infected or co-infected mice. We used RT-pre-amp-dPCR to detect and measure the presence of *B. afzelii* mRNA and TBEV RNA in both the engorged larvae and the nymphs post-molting. Our findings showed that in the group with *B. afzelii*, a significant majority (73%) of the larvae had *B. afzelii* mRNA (Fig. S2a). However, this percentage varied in other groups: only 15% in the S1 group, 40% in S2, and 30% in the co-inf. group showed the presence of *B. afzelii* mRNA (Table 5). Intriguingly, none of the engorged larvae in the TBEV, S1, S2, and co-inf. groups tested positive for TBEV RNA (Fig. S2b). Only one nymph showed the presence of TBEV RNA in the TBEV group and in the co-inf. group with both pathogens (Fig. S2d).

**TABLE 5:** RT-pre-amp-dPCR quantification in ticks targeting *Borrelia afzelii* mRNA and TBEV RNA

% of infection	Engorged larvae		Nymph after molt	
	*B. afzelii* mRNA	TBEV RNA	*B. afzelii* mRNA	TBEV RNA
*B. afzelii*	73%^*a*^ (11/15)	nd^*b*^	30% (3/10)	nd
TBEV	nd	0% (0/15)	nd	10% (1/10)
Super-infection 1	15% (3/20)	0% (0/20)	20% (1/5)	0% (0/5)
Super-infection 2	40% (6/15)	0% (0/15)	0% (0/10)	0% (0/10)
Co-infection	30% (6/20)	0% (0/20)	40% (4/10)	10% (1/10)

^*a*^ The number of positive ticks/total analyzed [percentage of infection (%)].

^*b*^ nd, no data.

## DISCUSSION

This study had two main objectives. The first objective was to create a co-infection model using C3H mice, involving both TBEV and *B. afzelii*. This was the initial step to assess the potential interactions between these pathogens in co-infected mice and then to investigate the transmission of these pathogens from co-infected mice to uninfected ticks.

Building upon our previous work with single infection models for *B. afzelii* (20) and TBEV (19) in C3H mice, we established a co-infection model. The outcomes of this model varied, depending on the timing of the co-infection. When mice were infected with TBEV 9 days after *B. afzelii* infection, TBEV symptoms worsened, and virus levels increased. Conversely, when mice were infected with TBEV twenty-one days after exposure to *B. afzelii*, they exhibited milder symptoms and lower mortality rates. Interestingly, co-infection with TBEV and *B. afzelii* resulted in mild symptoms and no fatalities.

Co-infection can result in various interactions between pathogens, leading to different outcomes (26). Extensive research on C3H mice has explored co-infections involving tick-borne pathogens such as *B. burgdorferi* s.s., *Ba. microti*, and *A. phagocytophilum*. These co-infections can significantly impact the body’s response and the overall disease outcome. For instance, co-infection of C3H mice with both *B. burgdorferi* s.s. and *Ba. microti* leads to higher spirochete levels in various organs, resulting in more severe and persistent Lyme disease symptoms, likely due to *Ba. microti*’s immunosuppressive effects and exacerbated inflammatory arthritis (16, 27 – 29). However, contradictory results were reported in a preliminary study when the pathogens followed independent pathways in C3H co-infected mice, and disease severity remained similar to that of single infections (30).

In this study, when comparing the outcomes of TBEV and *B. afzelii* infection groups to those of S1, S2, and co-infection groups, we made an unexpected discovery. Notably, not all mice in the TBEV group tested positive, in contrast to the various infection groups. This result was surprising, given that a previous experiment with the same infectious dose had resulted in infection and severe symptoms in all mice (19). The discrepancy may be attributed to differences in the timing of infection, potentially influenced by the age of the mice at the time of TBEV infection, as they were 8 weeks old instead of 6 weeks old (14).

Furthermore, the mice in the S2 group exhibited more severe symptoms and higher mortality rates compared to those in the S1 and co-inf. groups. Digital PCR analysis revealed a high viral RNA concentration (10^6^ copies/µL) in the brains of mice in the S2 group and (averaging 10^5^ copies/µL) in the S1 and co-inf. groups, indicating a preference for the central nervous system (CNS). Notably, this study presents the first evidence of *B. afzelii*’s ability to infect the brain. This infection was observed in a single mouse from the *B. afzelii* group as well as in both S1 and S2 groups. It is widely acknowledged that *B. garinii* and *B. burgdorferi* s.s. are the primary *Borrelia* species responsible for CNS infections. Specifically, *B. garinii* is commonly found in the cerebrospinal fluid of European patients, while *B. burgdorferi* s.s. is associated with acute Lyme neuroborreliosis in US patients. Although the precise mechanism remains unclear, it is plausible that hematogenous dissemination provides a viable route for spirochetes to enter the CNS (31). This hypothesis was supported by a recent study where *B. burgdorferi* s.s. was found to colonize the dura mater in C3H mice infected with 10^6^ bacteria (32). A similar finding has been described in a patient with Lyme disease, where *B. burgdorferi* was identified in the brain and spinal cord tissue (33). However, further research is needed to validate these findings.

As anticipated, co-infected mice exhibited nearly identical levels of *B. afzelii* DNA in their joints across different groups. Conversely, the detection and subsequent quantification of TBEV RNA in the joints yielded unexpected results. When comparing the levels of TBEV RNA pathogens between the TBEV group and the S1, S2, and co-inf. groups, particularly elevated levels were found in the S1 and S2 groups. Unfortunately, we could not compare these results to other studies because joint testing for TBEV is uncommon, as it is not considered a target organ. This trend also extended to the ear and bladder, where increased levels of TBEV RNA were detected in the S1, S2, and co-inf. groups compared to the TBEV group. These findings suggest that the viral load of TBEV increases in the joint, ear, and bladder in various super-infected and co-infected C3H mice groups compared to the TBEV group, while the *B. afzelii* load remains relatively constant. These results clearly show that variability in the time gap between infections impacts pathogen interactions. For example, the first pathogen may gain a competitive advantage by monopolizing host resources before the arrival of the second invader. Alternatively, the first pathogen may induce cross-reactive host immune responses that reduce the effectiveness of the subsequent pathogen (26).

In contrast, another study examining bacterial co-infection with *B. afzelii* and *A. phagocytophilum* showed an increase in Lyme spirochete load in the ears, heart, and skin of co-infected C3H mice, with no change in *A. phagocytophilum* load (15). In humans, concomitant infection with both *B. burgdorferi* s.s. and *Ba. microti* has been reported to synergistically increase the severity of Lyme disease (34).

Hence, our experiment implies a synergistic interaction between TBEV and *B. afzelii* in the murine host, resulting in the development of severe TBEV symptoms. However, our results also yielded an unexpected outcome: mice co-infected simultaneously with both pathogens in the co-inf. group displayed only mild symptoms and did not succumb to the infections. This contradicted our initial expectations, as we had hypothesized that TBEV would dominate over *B. afzelii*. Moreover, among the super-infection and co-inf. groups, S1 had lower mortality rates, despite having similar pathogen levels in their organs. Mice in both S1 and co-inf. groups exhibited fewer symptoms than expected for TBEV infection, suggesting an antagonistic interaction between the two pathogens. However, the specific nature of this competition remains unclear.

In the scientific literature, it is widely acknowledged that competition arises in cases of multi-infection (35). Such interactions can influence various factors, including the abundance of different pathogen species, pathogen loads, transmission, and virulence. Pathogens can compete with each other through various mechanisms, including the production of toxins, as seen with *Enterobacter* bacteria conferring resistance to *Plasmodium falciparum* in mosquitoes (36). Competition can also result in growth inhibition, as evidenced by instances of resource competition-driven anemia caused by helminths (35). Another example can be found in the work of Purnell et al., who observed that the presence of both *A. phagocytophilum* and *Babesia divergens* in calves simultaneously tended to suppress *Ba. divergens* infection, but if *Ba. divergens* appeared several weeks after *A. phagocytophilum*, babesiosis worsened (37).

The host immune system’s response may also contribute to this competition. Many co-infection studies employ C3H models involving *B. burgdorferi* s.s. and *A. phagocytophilum*. These studies investigate how these two infections collectively impact the immune response and the development of Lyme arthritis. They have revealed that co-infection in mice results in alterations in certain immune interleukins (ILs), including decreased levels of IL-12, interferon gamma, and tumor necrosis factor-alpha, and increased levels of IL-6. Additionally, the activation of macrophages diminishes during simultaneous infection, rendering them less effective at phagocytizing pathogens. Moreover, simultaneous infection enhances pathogen transmission from the host to ticks by modulating immunity (15, 16). Studying the immune response in co-infected mice will help elucidate the synergistic and/or antagonistic interactions that appear to occur between *B. afzelii* and TBEV.

The second objective of our experiment was to investigate whether ticks could acquire these pathogens by feeding on either single or co-infected mice. Our focus was on evaluating the efficiency of pathogen acquisition and transmission from mice that were simultaneously infected to pathogen-free ticks. While we successfully detected the presence of *B. afzelii* in fully engorged larvae, there was no evidence of TBEV RNA based on our dPCR analysis. Interestingly, during the process of *trans* stadial transmission, we observed that only one nymph became co-infected with both pathogens after feeding on a mouse from the co-inf. group. However, a small number of nymphs still tested positive in the *B. afzelii* group. One possible explanation for TBEV’s failure to transmit may be the low dose of 10^2^ PFU in the bloodstream, which appeared insufficient for transmitting TBEV from infected mice to ticks. Another plausible explanation could be that the RNA copies of TBEV in our infected ticks fell below the detectable limit (19).

### Conclusion

In summary, this study successfully demonstrated that C3H mice can be super-infected or co-infected with both *B. afzelii* and TBEV. Given the value of C3H mice as a model for tick-borne diseases, it is imperative to further explore their immune response. Investigating how the immune system responds in the presence of both pathogens in C3H mice will enhance our understanding of the interplay between TBEV and *B. afzelii*. For future research, it is essential to investigate the transmission of pathogens between co-infected mice and uninfected ticks. Currently, this co-infection model does not effectively infect ticks with TBEV (either single-infected or co-infected). A potential solution may involve increasing the TBEV dose used for infecting mice, although this could lead to heightened symptom severity, potentially deviating from natural conditions. Understanding the consequences of co-infection is crucial, especially given the rising prevalence of tick-borne diseases such as Lyme disease and TBE. Individuals co-infected with both pathogens may experience heightened symptoms due to TBEV and/or *B. afzelii*. Therefore, we must strive to gain a comprehensive understanding of the implications of co-infection by using and refining animal model systems. This endeavor will contribute to advancing knowledge and addressing gaps in our understanding of tick-borne diseases.

## SUPPLEMENTAL MATERIAL

Figure S1 iai.00249-24-s0001.tiff Detection of *Borrelia afzelii* proteins.

Figure S2 iai.00249-24-s0002.tiff
*Borrelia afzelii* mRNA and TBEV RNA quantification in ticks by RT-preamp-digital PCR.

Supplemental legends iai.00249-24-s0003.docx Legends for Fig. S1 and S2**.**

ASM does not own the copyrights to Supplemental Material that may be linked to, or accessed through, an article. The authors have granted ASM a non-exclusive, world-wide license to publish the Supplemental Material files. Please contact the corresponding author directly for reuse.
