# Supplementary figures and images for: Co-infection dynamics of B. afzelii and TBEV in C3H mice: insights and implications for future research
Source: Infect Immun. 2024 Jul 11;92(8):e00249-24. doi: 10.1128/iai.00249-24 (PMC11320977; doi:10.1128/iai.00249-24)

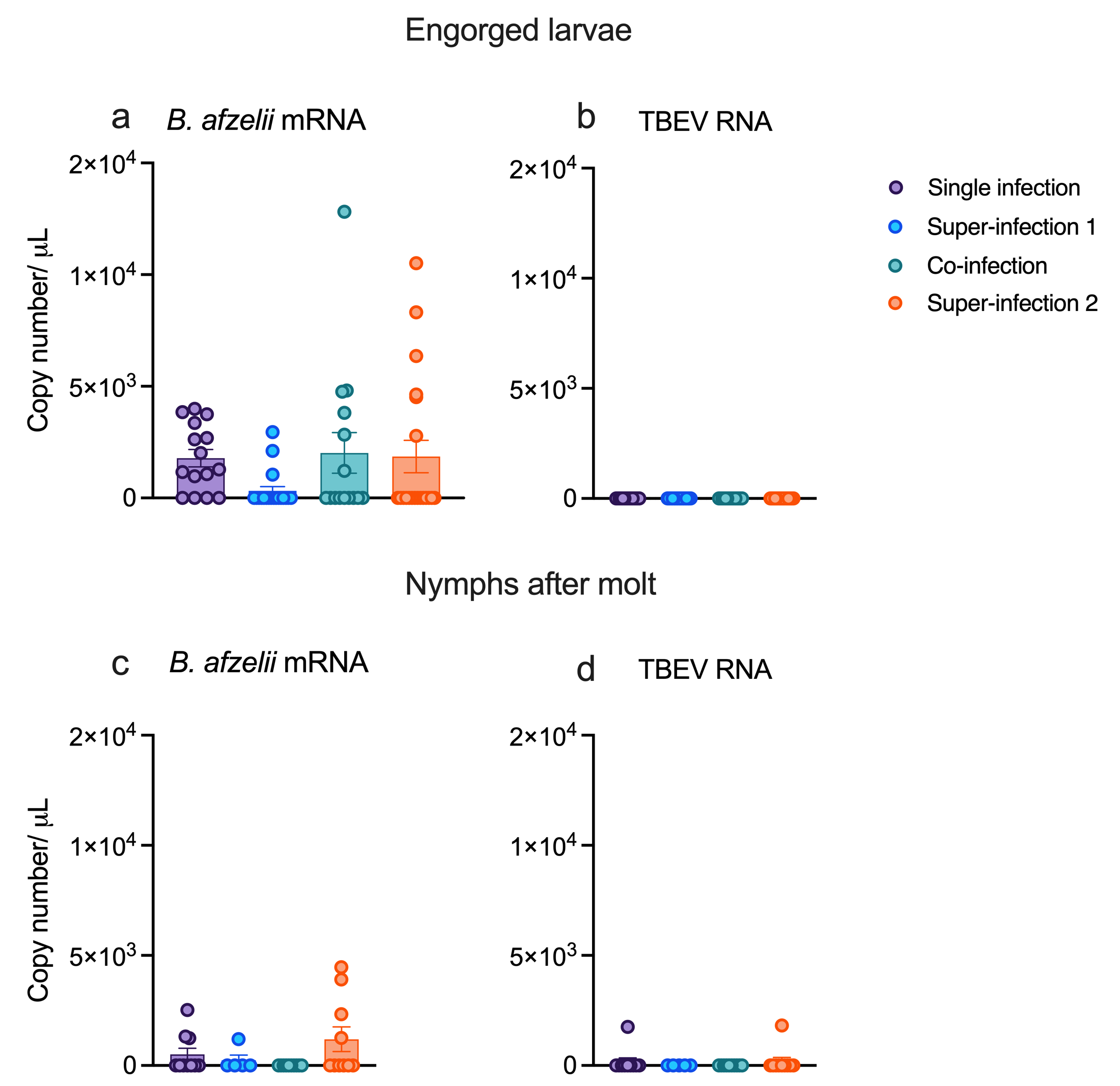

Supplement: Figure S2 — Borrelia afzelii mRNA and TBEV RNA quantification in ticks by RT-preamp-digital PCR. [file iai.00249-24-s0002.tiff]
