# Supplementary material for: Co-infection dynamics of B. afzelii and TBEV in C3H mice: insights and implications for future research
Source: Infect Immun. 2024 Jul 11;92(8):e00249-24. doi: 10.1128/iai.00249-24 (PMC11320977; doi:10.1128/iai.00249-24)
Supplement: Supplemental legends — Legends for Fig. S1 and S2. [file iai.00249-24-s0003.docx]

**Supplementary materials**

**Figure S1. Detection of *Borrelia afzelii* proteins.** The identification of *Borrelia* proteins involved western blot analysis using sera from mice experimentally infected with *B. afzelii* eight days post-TBEV infection.

**Figure S2**. ***Borrelia afzelii* mRNA and TBEV RNA quantification in ticks by RT-preamp-digital PCR.** *Borrelia afzelii* mRNA and TBEV RNA in engorged larvae are labelled ‘a’ and ‘b’, respectively. While *Borrelia afzelii* mRNA and TBEV RNA nymphs after molt are labelled ‘c’ and ‘d’, respectively. Means and standard errors are shown.
